# Pre-Conception Interventions for Subfertile Couples Undergoing Assisted Reproductive Technology Treatment: Modeling Analysis

**DOI:** 10.2196/19570

**Published:** 2020-11-23

**Authors:** Régine Steegers-Theunissen, Annemieke Hoek, Henk Groen, Annelies Bos, Grada van den Dool, Marieke Schoonenberg, Jesper Smeenk, Eva Creutzberg, Loes Vecht, Luc Starmans, Joop Laven

**Affiliations:** 1 Department of Obstetrics and Gynaecology Erasmus MC University Medical Center Rotterdam Netherlands; 2 Department of Obstetrics and Gynaecology University Medical Center Groningen University of Groningen Groningen Netherlands; 3 Department of Epidemiology University Medical Center Groningen University of Groningen Groningen Netherlands; 4 Department of Reproductive Medicine and Gynecology University Medical Centre Utrecht Utrecht Netherlands; 5 Department of Obstetrics and Gynaecology Albert Schweitzer Hospital Zwijndrecht Zwijndrecht Netherlands; 6 Nij Geertgen Centre for fertility Elsendorp Elsendorp Netherlands; 7 Department of Obstetrics and Gynaecology Elisabeth-TweeSteden Hospital Tilburg Tilburg Netherlands; 8 Department of Gynaecology Ferring BV Hoofddorp Amsterdam Netherlands; 9 KPMG Amsterdam Netherlands

**Keywords:** fertility, periconception, pregnancy chance, Smarter Pregnancy, cost-effectiveness, nutrition, obesity, IVF treatment, mobile and web-based lifestyle apps

## Abstract

**Background:**

Approximately 1 in 7 couples experience subfertility, many of whom have lifestyles that negatively affect fertility, such as poor nutrition, low physical activity, obesity, smoking, or alcohol consumption. Reducing lifestyle risk factors prior to pregnancy or assisted reproductive technology treatment contributes to the improvement of reproductive health, but cost-implications are unknown.

**Objective:**

The goal of this study was to evaluate reproductive, maternal pregnancy, and birth outcomes, as well as the costs of pre-conception lifestyle intervention programs in subfertile couples and obese women undergoing assisted reproductive technology.

**Methods:**

Using a hypothetical model based on quantitative parameters from published literature and expert opinion, we evaluated the following lifestyle intervention programs: (1) Smarter Pregnancy, an online tool; (2) LIFEstyle, which provides outpatient support for obese women; (3) concurrent use of both Smarter Pregnancy and LIFEstyle for obese women; (4) smoking cessation in men; and (5) a mindfulness mental health support program using group therapy sessions. The model population was based on data from the Netherlands.

**Results:**

All model-based analyses of the lifestyle interventions showed a reduction in the number of in vitro fertilization, intracytoplasmic sperm injection, or intrauterine insemination treatments required to achieve pregnancy and successful birth for couples in the Netherlands. Smarter Pregnancy was modeled to have the largest increase in spontaneous pregnancy rate (13.0%) and the largest absolute reduction in potential assisted reproductive technology treatments. Among obese subfertile women, LIFEstyle was modeled to show a reduction in the occurrence of gestational diabetes, maternal hypertensive pregnancy complications, and preterm births by 4.4%, 3.8%, and 3.0%, respectively, per couple. Modeled cost savings per couple per year were €41 (US $48.66), €360 (US $427.23), €513 (US $608.80), €586 (US $695.43), and €1163 (US $1380.18) for smoking cessation, mindfulness, Smarter Pregnancy, combined Smarter Pregnancy AND LIFEstyle, and LIFEstyle interventions, respectively.

**Conclusions:**

Although we modeled the potential impact on reproductive outcomes and costs of fertility treatment rather than collecting real-world data, our model suggests that of the lifestyle interventions for encouraging healthier behaviors, all are likely to be cost effective and appear to have positive effects on reproductive, maternal pregnancy, and birth outcomes. Further real-world data are required to determine the cost-effectiveness of pre-conception lifestyle interventions, including mobile apps and web-based tools that help improve lifestyle, and their effects on reproductive health. We believe that further implementation of the lifestyle app Smarter Pregnancy designed for subfertile couples seeking assistance to become pregnant is likely to be cost-effective and would allow reproductive health outcomes to be collected.

## Introduction

Many couples who undergo fertility treatment have multiple lifestyle risk factors that reduce their chances of becoming pregnant [1]. Female lifestyle risk factors such as smoking, alcohol use, and poor diet are inversely associated with fecundity and time to pregnancy, and the effects of these factors increase with increasing BMI [2]. Chronic stress or high anxiety levels reduce fecundability, and high depression scores are also associated with subfertility [3]. Furthermore, evidence is accumulating that poor nutrition, stress, drug use, infection, or exposure to environmental chemicals during prenatal development has a life-long impact on offspring health as shown by the Developmental Origins of Health and Disease paradigm [4]. Obese pregnant women are more likely to have gestational diabetes, hypertensive complications, premature delivery, higher risk of cesarean delivery, and fetal death [5]. Obese men are more likely than men of normal weight to have lower sperm quality [6].

Despite the evidence available, weight-loss interventions in overweight or obese couples prior to fertility treatment remain controversial, as there is limited evidence that these interventions increase the chance of a live birth or reduce pregnancy complications [7,8]. Two well-conducted randomized controlled trials [9,10], however, show that spontaneous conceptions significantly increased with pre-conception weight-loss lifestyle interventions in obese subfertile women and led to lower number of fertility treatments, though neither trial showed an increase in assisted reproductive technology treatment-dependent pregnancies.

Health care budgets are increasingly under pressure due to noncommunicable diseases associated with an aging population, scarcity in the workforce, and rising costs of novel medical technologies. It is, therefore, key that lifestyle interventions aimed at subfertile couples improve reproductive outcomes and in a cost-effective manner.

The aim of this evaluation was to estimate and model the impact of pre-conception lifestyle interventions on the likelihood of the occurrence of pregnancy, and maternal pregnancy and birth outcomes after fertility treatment in subfertile couples and in subfertile obese women, as well as to assess whether these lifestyle interventions are cost-effective.

## Methods

### Overview

We created a hypothetical model to estimate the effectiveness of pre-conception lifestyle interventions on reproductive, pregnancy, and birth outcomes and potential cost savings in subfertile couples, including a subgroup of subfertile obese women, undergoing in vitro fertilization (IVF), intracytoplasmic sperm injection (ICSI), or intrauterine insemination (IUI) treatment.

### Lifestyle Factors Relevant to Fertility Care

Modifiable factors most relevant to fertility treatment, reproductive health, and pregnancy complications with compelling quantifiable clinical published evidence were women’s BMI (a marker of health and lifestyle), diet (nutrition intake), physical activity, smoking cessation, and stress [2,9,11-23].

### Pre-conception Lifestyle Interventions

Five lifestyle interventions matching the selected lifestyle factors were selected by the fertility experts for inclusion in the cost-effectiveness model (Table 1). These programs included support for changing lifestyle factors deemed most relevant to pre-conception care that were outlined above. The programs used in our model were Smarter Pregnancy [12,20], and LIFEstyle [9,11]. We also modeled smoking cessation in men by using analysis of IVF and ICSI outcomes in men who smoke and men who do not smoke [24], and mindfulness-based mental health support for couples undergoing IVF [16].

**Table 1:** Lifestyle interventions selected for cost-effectiveness modeling.

Name	Model target population	Description
Smarter Pregnancy intervention [12,20]	Women aged 25-44 years trying to conceive, comprising subfertile couples seeking medical assistance to conceive (IVF^a^/ICSI^b^) and fertile couples	Provides 26 weeks of individual online coaching and information via smartphone tailored to improve nutrition and lifestyle during the pre-conception and pregnancy period in order to improve the health of the reproductive population and subsequent generations
LIFEstyle intervention [9,11]	Obese subfertile women with BMI >29 kg/m^2^ seeking fertility treatment	Provides 6 outpatient visits (each 30 minutes long) and 4 telephone consultations (15 minutes) during a 24-week period to provide motivation and support for nutrition (energy restriction of approximately 500 kCal/day) and exercise strategies (10,000 steps per day, 2-3 moderate vigorous exercise sessions per week for weight loss in obese women (BMI >29 kg/m^2^) seeking fertility treatment
Combined Smarter Pregnancy and LIFEstyle intervention [9,11,12,20]	Combination of the 2 target audiences mentioned above	Providing both Smarter Pregnancy and LIFEstyle support for obese women (the remaining couples were modeled to receive the Smarter Pregnancy intervention only)
Smoking cessation [24]	Subfertile men who smoke	Comparison of IVF and ICSI outcomes in male smokers and non-smokers from couples seeks reproductive assistance
Mindfulness support [16]	Subfertile women undergoing their first IVF or ICSI cycle	Comparison of IVF outcomes in couples either receiving or not receiving group sessions to teach stress reduction through a mindfulness-based intervention while undergoing IVF treatment

^a^IVF: in vitro fertilization.

^b^ICSI: intracytoplasmic sperm injection.

### Model Population and Treatment Policies

The model population comprised couples living in the Netherlands seeking fertility treatment, representing a general subfertile population including a subgroup of obese women. The prevalence of subfertility in men and women living in the Netherlands aged 25-45 years was 0.7% and 2.2%, respectively, representing approximately 15,000 men and 46,000 women who are subfertile (Multimedia Appendix 1). Based on available published data [25,26], we calculated that there were 5400 subfertile obese women, 3200 smoking men, and 13,700 women undergoing their first IVF or ICSI cycle. Based on clinical experience, we assumed approximately 24% of the couples living in the Netherlands seeking assisted reproductive technology treatment received IVF treatment (mean 1.5 cycles), 16% received ICSI (mean 1.5 cycles) in total per couple, and 60% received IUI (mean 3.0 cycles). We did not include couples seeking ovulation induction in our model.

### Modeling Clinical Outcomes

For each pre-conception lifestyle intervention program, the published data were reviewed to determine input parameters for the model to estimate the intervention's impact on chance of a spontaneous ongoing pregnancy (a viable pregnancy at week 12), number of IVF, ICSI, and IUI treatments, as well as on pregnancy complications— gestational diabetes, gestational hypertensive complications, and preterm delivery—expressed per couple per year. For each lifestyle intervention, the model only included the fertility treatments and known complications from the lifestyle intervention according to high quality evidence.

### Modeling Potential Cost Savings

**Figure 1:**
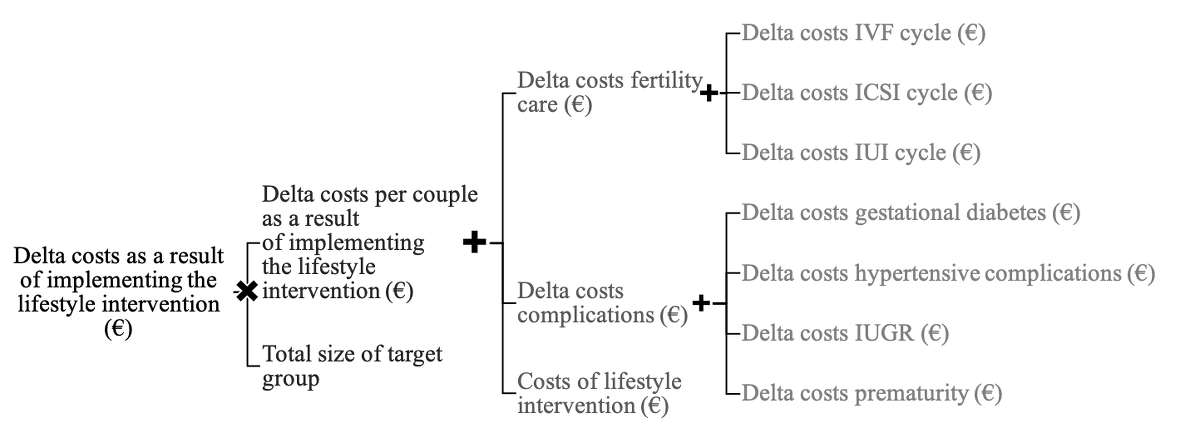
General structure of the cost-effectiveness model for each lifestyle intervention. IUGR: intrauterine growth restriction; IUI: intrauterine insemination; IVF: in vitro fertilization; ICSI: intracytoplasmic sperm injection.

The general structure of the cost-effectiveness model is depicted in Figure 1. The cost-effectiveness evaluation was performed from a health care perspective and included direct medical costs of the lifestyle intervention, fertility treatments, medication, and any resulting pregnancy. The model (business case) was designed to estimate the cost impact of the selected lifestyle interventions on fertility and obstetric care per subfertile couple, expressed as the difference in costs per patient or per couple and the overall cost difference per year in the Netherlands.

### Model Input Parameters

The effects of the respective interventions on each of the selected lifestyle factors were modeled according to available published literature or expert opinion agreed by consensus. Costs for ovulation induction prior to IUI were not included as part of the cost-effectiveness model. Where published data existed, previously estimated costs of pregnancy complications, such as fetal growth restriction, gestational diabetes, gestational hypertensive complications (including preeclampsia), and premature birth, were used.

The general costs for assisted reproductive technology and pregnancy are presented in Multimedia Appendix 1. Costs, indexed to 2016 price levels, for pregnancy, birth and admission until 6 weeks postpartum in singleton and multiple pregnancies conceived after IVF were estimated.

Specific parameters related to each pre-conception lifestyle intervention are presented in Multimedia Appendix 2 including costs of the intervention, the estimated costs of pregnancy outcomes, chances of spontaneous pregnancy, intrauterine growth restriction, and gestational diabetes or hypertension. For each lifestyle intervention that was modeled, the estimated impact on fertility treatment or pregnancy outcomes were only included if there were data to support effects associated with that specific lifestyle intervention.

## Results

### Modeled Impact of Lifestyle Interventions on Clinical Outcomes

The modeled impact of lifestyle interventions on fertility outcomes, including the reductions in assisted reproduction and pregnancy complications, are presented in Tables 2 and 3. Each of the 5 modeled lifestyle interventions showed a reduction in the number of IVF and ICSI treatments per couple required to achieve a successful ongoing pregnancy. Smarter Pregnancy in the subfertile couples and LIFEstyle in subfertile obese women resulted in 10,800 (23.4%) and 1100 (20.0%) fewer IUI cycles, respectively, among the modeled population for the Netherlands than if no lifestyle intervention was used. Overall, Smarter Pregnancy was modeled to have both the largest increase in the number of spontaneous pregnancies (6000 additional spontaneous pregnancies among the modeled population, which is a 13% reduction in the number of couples who require assisted reproductive technology treatment) and the largest reduction in potential number of assisted reproductive technology treatments per couple (4.7% for in vitro fertilization, 3.1% for intracytoplasmic sperm injection, and 23.4% for intrauterine insemination). Smarter Pregnancy was associated with a reduction of fetal growth restriction by 2.6%, and LIFEstyle was associated with a reduction of gestational diabetes (4.4%), gestational hypertensive complications (3.8%), and preterm delivery (3.0%). There were no published data regarding the effects of smoking cessation or mindfulness on the likelihood of achieving spontaneous pregnancies, or on fetal growth restriction, gestational diabetes, hypertensive, or premature birth pregnancy complication.

**Table 2:** The effect of each lifestyle intervention on assisted reproductive technology clinical outcomes modeled for the Netherlands.

Intervention	Modeled changes in clinical results (per year)			
	Spontaneous pregnancies, n (%)^a^	IVF^b^ treatments, n (%)	ICSI^c^ treatments, n (%)	IUI^d^ treatments, n (%)
Smarter Pregnancy (SlimmerZwanger)	+6000 (13.0)	–2200 (–4.7)	–1400 (–3.1)	–10,800 (–23.4)
LIFEstyle	+500 (9.9)	–600 (–11.3)	–1000 (–18.9)	–1100 (–20.0)
Smarter Pregnancy + LIFEstyle^e^	+6000 (13.0)	–2500 (–5.5)	–2300 (–5.0)	–10,800 (–23.4)
Smoking cessation (men only)	—^f^	–300 (–0.8)	–100 (–0.4)	—
Mental health/ mindfulness	—	–1600 (–11.8)	–100 (–0.9)	—

^a^Percentage has been calculated as the proportional change in the number of events (spontaneous pregnancy or assisted reproductive technology treatment resulting in pregnancy) for the model population per intervention.

^b^IVF: in vitro fertilization.

^c^ICSI: intracytoplasmic sperm injection.

^d^IUI: intrauterine insemination.

^e^Only for the subset of women with BMI over 29 kg/m^2^.

^f^No published data found in literature searches.

**Table 3:** Effect of each lifestyle intervention on clinical outcomes of pregnancy complications modeled for the Netherlands (modeled per year).

Intervention	Modeled changes in clinical results (per year)			
	IUGR^a^, n (% change)^b^	Gestational diabetes, n (% change)	Hypertensive complications, n (% change)	Decreased number of premature births, n (% change)
Smarter Pregnancy (*SlimmerZwanger*)	–1200 (–2.6)	—^c^	—	—
LIFEstyle	—	–200 (–4.4)	–200 (–3.8)	–200 (–3.0)
Smarter Pregnancy + LIFEstyle	–1200 (–2.6)^d^	–200 (–4.4)^d^	–200 (–3.8)^d^	–200 (–3.0)^d^
Smoking cessation (men only)	—	—	—	—
Mental health/ mindfulness	—	—	—	—

^a^IUGR: intrauterine growth restriction.

^b^Percentage has been calculated as the proportional change in the number of events (pregnancy complications) for the model population per intervention).

^c^No published data found in literature searches.

^d^Only for the subset of women with BMI over 29 kg/m^2^.

### Estimated Cost-Savings of Lifestyle Interventions and Sensitivity Analysis

A summary of the estimated cost savings for each lifestyle intervention is presented in Table 4. The lifestyle intervention that was modeled to have the highest cost saving was LIFEstyle, with an estimated saving of €1163 (US $1380.18 at the time of publication) per couple; however, this intervention is specifically for subfertile obese women. The combination of Smarter Pregnancy and LIFEstyle, for which obese women would have access to both Smarter Pregnancy and LIFEstyle and all other subfertile couples would use Smarter Pregnancy only, was modeled to save €586 (US $695.43) per couple. Across the entire potential target group in the Netherlands, the greatest financial saving would be achieved with the combination program Smarter Pregnancy and LIFEstyle (€27 million, approximately US $32 million), followed by Smarter Pregnancy (€24 million, approximately US $28.4 million) alone. The lifestyle intervention of smoking cessation for men represented the lowest cost saving €41 (US $48.66) per couple in the Netherlands.

**Table 4:** Estimated cost savings per year of lifestyle interventions (business cases) on assisted reproductive technologies and reduction of pregnancy complications modeled for the Netherlands (an exchange rate of approximately €1=US $1.19 is applicable at the time of publication).

Intervention	Estimated cost benefit per couple^a^ (least favorable, most favorable scenario)	Total target group (prevalence)	Total annual target group (incidence)	Estimated overall cost saving (least favorable, most favorable scenario) per year^a^	Estimated total assisted reproductive technology cost saving per year (least favorable, most favorable scenario)^b^
Smarter Pregnancy (*SlimmerZwanger*)	€513 (€100, €2200)	46,000	1191	€24 M (€4.6 M, €101.2 M)	€6 M (€1.2 M, €26.2 M)
LIFEstyle	€1163 (€900, €1600)	5400	1391	€6 M (€4.9 M, €8.6 M)	€1.6 M (€1.3 M, €101 M)
Smarter Pregnancy + LIFEstyle	€586 (€100, €2200)	46,000	1191	€27 M (€4.6 M, €101 M)	€7 M (€4.6 M, €26.4 M)
Smoking cessation (men only)	€41 (€–20, €170)	3200	826	€0.130 M (€–0.064 M, €0.54 M)	€0.034 M (€–0.017 M, €0.140 M)
Mental health/ Mindfulness	€36 (€–190, €500)	13,700	3551	€4.9 M (€–2.6 M, €6.9 M)	€1.3 M (€–0.7 M, €1.8 M)

^a^Overall cost savings include all medical intervention costs for complications.

^b^Includes cost savings for assisted reproductive technology procedures that would no longer be necessary.

## Discussion

### Principal Results

Using a model population based on subfertile couples and subfertile obese women living in the Netherlands undergoing IVF, ICSI, or IUI, we developed a hypothesis-based model using quantitative parameters from published literature and expert opinion to explore how 5 selected lifestyle intervention programs, each using different approaches and targets, can potentially improve the chances of spontaneous pregnancy, reduce the number of cycles of IVF, ICSI, or IUI treatments, and reduce the chance of adverse maternal pregnancy and birth outcomes. As part of the analysis our model also assessed potential cost savings of the selected lifestyle intervention programs for the model population. In order to estimate the cost savings, we calculated the estimated cost savings per couple for each of the selected lifestyle intervention programs. Multiplying this number with the size of the target group of the selected lifestyle intervention program yielded the total estimated cost savings. Cost savings per couple were modeled for each of the lifestyle programs taking into account the cost savings achieved by reducing the volume of fertility care, as well as cost savings achieved due to a reduction of pregnancy complications. Estimated costs of the lifestyle intervention were subsequently subtracted from these savings, in order to achieve the total cost savings of the lifestyle intervention. For each lifestyle intervention, the model only included the fertility treatments and complications that the lifestyle intervention was known to have an effect upon using available evidence.

Modifiable lifestyle factors were selected based on their positive associations with the likelihood of spontaneous conception or successful fertility treatment and thus would be most relevant for evaluating in terms of potential cost saving. Age was considered to be important but not modifiable at the moment of the health care visit. In men, moderate alcohol consumption, caffeine intake, and scrotal temperature were considered less relevant lifestyle factors and, therefore, were not included in our model. Thus, the most important modifiable factors were maternal BMI (used as a surrogate marker of health and lifestyle), diet, physical activity, smoking, and stress. These factors had sufficient published quantitative data regarding their impact on reproductive health, fertility treatment, and pregnancy complications to develop our model and have also used by others [9,11,12,16,20,24].

In our model, both Smarter Pregnancy and LIFEstyle increased the number of spontaneous pregnancies by 13.0% and 9.9% per couple, respectively, compared with no lifestyle intervention. This supports data from another study that showed that a 1-point increase in a pre-conception dietary risk score was associated with 65% increased chance of ongoing pregnancy [27] and a “Mediterranean”-style diet is likely to improve IVF and ICSI treatment success with an increased probability of pregnancy (odds ratio 1.4, 95% CI 1.0-1.9) [28]. Similarly, in our model, these 2 lifestyle interventions decreased the number of IVF, ICSI, and IUI cycles required for a successful pregnancy. Although there were no data available for the effect of smoking cessation in men and mindfulness mental health support on spontaneous pregnancies or number of IUI treatments, our model suggests that both may decrease the number of IVF and ICSI treatments required for a successful pregnancy.

Our model also showed that validated lifestyle interventions may contribute to a reduction of pregnancy complications, including fetal growth restriction, gestational diabetes, hypertensive complications and premature births. Published clinical data about fetal growth restriction were only available to model Smarter Pregnancy, for which there was a 2.6% decreased occurrence of fetal growth restriction per couple. Indeed, the evidence for pre-conception nutrition associated with birth weight is compelling, with studies advocating “Mediterranean”-style diets high in fruit, vegetables, vegetable oil, fish, pasta, and rice as well as lower consumption of meat and potatoes [28,29]. For example, the size of the embryo represented by the crown-rump length is improved by an energy-rich nutritious diet (effect estimate 1.62, 95% CI 0.52-2.72; *P*<.05) [29]. Moreover, pre-conception diets with increased pre-conception omega-3 polyunsaturated fatty acid intake from fruit and vegetables was associated with improved embryo morphology (linear regression coefficient β=0.6, *P*≤.05) [30]. Similarly, a “traditional Western” diet (high intake potatoes, mayonnaise and other fatty sauces, meat products, refined grains, sugar, and confectionary) was associated with an sperm DNA damage (linear regression coefficient β=13.25, *P*=.01), whereas a “health conscious” diet (high intakes of fruit, vegetables, fish and other seafood, whole grains and legumes) was inversely associated with sperm DNA damage (β=–2.81, *P*=.05) [31].

In contrast, published clinical data on gestational diabetes, hypertensive complications, and premature births were only available to model LIFEstyle, the pre-conception intervention to help obese women lose weight prior to and during early pregnancy. We modeled that implementation of the LIFEstyle program for obese women in the Netherlands seeking reproductive assistance may result in 4.4% lower chance of gestational diabetes, 3.8% lower chance of hypertensive complications, and 3.0% chance of premature birth. In subanalyses of the LIFEstyle study, women in the intervention group significantly reduced their consumption of sugary drinks and savory snacks, as well as increased their physical activity [32], and reduced the likelihood of developing metabolic syndrome [33]. A recent meta-analysis has shown that healthy diets (Mediterranean Diet, Dietary Approaches to Stop Hypertension diet and Alternate Healthy Eating Index diet) were associated with 15%-38% reduced relative risk of gestational diabetes, and compared with no physical activity, any prepregnancy or early pregnancy physical activity was associated with 30% and 21% reduced odds of gestational diabetes, respectively [34].

Our model also showed that lifestyle intervention programs are cost-effective to improve the chances of pregnancy [2]. It should be noted, however, that our estimated cost savings are conservative, as we did not include the cost of ovulation induction in our model. Moreover, we used conservative data regarding the number of subfertile men and women in the Netherlands. In our model, LIFEstyle has the greatest cost saving of €1163 (US $1380.18) per couple, which was similar to a cost analysis of the LIFEstyle study showed an overall saving of €1278 (US $1512.92) per couple [17].

### Limitations

Inherent to the model-based approach using existing literature and expert opinion input data, we show results which may not completely reflect the real-world. Moreover, we did not model the costs for ovulation induction as it has a high success rate for anovulatory infertility. Although our model was based on couples living in the Netherlands, it nevertheless reflects couples seeking fertility assistance in other wealthy developed countries, such as those in Western Europe, North America, and Australia [35]. Published literature pertaining some pregnancy outcomes was not available, which necessitated assumptions being made. The costs of fertility treatment vary greatly depending on country and assisted reproductive technology procedures used, and the costs of some procedures are uncertain. We modeled the potential impact on reproductive and cost of fertility treatment rather than collecting real-world data. Although every effort was made to include evidence-based input parameters, the prevalence of subfertility among men and women living in the Netherlands may have been underestimated in our model. However, by using a conservative estimate of the number, the results of our model are also conservative, meaning that it is possible that there could be greater overall reductions in the number of IVF, ICSI, and IUI treatments required, fewer pregnancy complications, and larger cost savings than we have reported here. It is important to stress that the estimated cost savings are likely an underestimation, as we have not modeled more indirect cost savings that likely are achieved by reducing pregnancy complications, such as for instance, a more expedited return to the workforce for the mother, as well as improved health of the newborn which likely will lead to fewer health care expenditures in the years following birth. In addition, we have not included cost savings that can reasonably be expected as a result of improved lifestyle in the mother and father, likely leading to fewer health care expenditures as well as potentially a greater contribution to the workforce (and therefore taxation).

In our model, we included a comparison of the effects of smoking on IVF and ICSI outcomes in men. To date, there have been no published studies on pregnancy outcomes regarding the use of smoking cessation apps or programs in couples seeking to become pregnant, therefore, we have likely underestimated the effects of smoking cessation on pregnancy outcomes and the cost implications of smoking cessation in terms of reducing the need for fertility assistance and pregnancy complications. In addition, the effects of second-hand smoke have not been considered in our model.

Another limitation is that the costs of fertility treatment vary greatly depending on country and procedures required, and the costs of some procedures are uncertain. The type of fertility treatment required would depend on the cause of subfertility, which we have not included or addressed in our model. Further investigations are required to understand to what extent lifestyle modifications can reduce the risk of pregnancy complications, as well as affect direct outcomes of fertility treatment.

### Comparisons With Prior Work

Our model input parameters were mostly based on good-quality evidence from published literature; however, as is inherently the case with hypothesis-based modeling, some assumptions were made. There have been 2 randomized controlled trials [9,10] that have investigated pre-conception lifestyle interventions to help obese women to lose weight. Although the study LIFEstyle [11] did not increase the healthy live birth rate, there was an increased rate of spontaneous pregnancies specifically among anovulatory women. A further exploratory analysis [13] suggested that a periconceptional decrease in BMI in obese subfertile women could lead to a decrease of the rates of hypertensive pregnancy complications and preterm birth; however, further randomized controlled trials are required to confirm these results. Similarly, in the second study, significantly more live births were achieved through spontaneous pregnancies in the weight reduction group (10.5%) than in the control group (2.6%; *P*=.009) [10]. Results from another randomized controlled trial, the UK Pregnancies Better Eating and Activity Trial (UPBEAT) [36], showed that specific dietary patterns in obese women in early pregnancy are linked to gestational diabetes; however, the early pregnancy UPBEAT intervention did not reduce the incidence of gestational diabetes in its cohort [37]. Nevertheless, a secondary analysis of UPBEAT suggested that women randomized to the UPBEAT intervention had healthier metabolic profiles than those who received standard care [38]. As such, overweight or obese couples are likely to benefit from pre-conception lifestyle modifications of improved nutrition and physical activity prior to fertility treatment in an effort increase the chances of spontaneous conception and to help reduce pregnancy complications of gestational diabetes, hypertensive complications, and preterm birth. Improving lifestyle during the periconception period (defined as at least 14 weeks before conception) and the first 10 weeks of pregnancy is likely to help alleviate the risk of several adverse birth outcomes, such as congenital malformations, fetal growth restriction and babies born small or large for gestational age, as well as maternal pregnancy complications such as gestational diabetes, hypertensive disorders, and premature delivery [11-13,27].

Our data are also supported by a previous model-based cost analysis for Smarter Pregnancy used by 793 subfertile women undergoing IVF treatment [2]. This program resulted in 86 additional pregnancies and saved €270,000 (approximately US $319,630) compared to usual care after 2 IVF cycles, with an incremental cost-effectiveness ratio of €–3050 (95% CI €–3960 to €–540; or approximately US $–3611, 95% CI US $–4688 to US $–639) per additional pregnancy. The largest cost saving was from avoided IVF treatment costs. Sensitivity analyses showed that Smarter Pregnancy would need to increase the ongoing pregnancy rate by at least 51% after 2 IVF cycles for cost saving. Thus, Smarter Pregnancy is potentially cost saving for subfertile couples after their first IVF treatment.

There have been no published randomized controlled trials specifically on pre-conception interventions to quit smoking or to reduce or stop alcohol consumption. Nevertheless, Smarter Pregnancy has assessed pre-conception healthy nutrition and lifestyle, including tailored advice based on questionnaire responses regarding smoking and alcohol consumption in both couples seeking fertility treatment, and couples conceiving spontaneously [12]. Importantly, the Smarter Pregnancy intervention has been the only study to show a positive correlation between women whose partners also made positive lifestyle modifications and achieving pregnancy [20]. In addition, Smarter Pregnancy was not tailored for obese women and their partner, but these couples also appreciated the program very much and showed comparable effectiveness. There remains a lack of randomized controlled trials in this area as first highlighted by a Cochrane review nearly a decade ago [39].

Current evidence on the effectiveness of internet or app-based interventions is limited, and further investigation is needed in order to fully appraise their impact on fertility outcomes such as pregnancy rate, as well as pregnancy complications and newborn health. A recent systematic review [40] looked into feasibility, acceptability, and effectiveness of mobile health lifestyle and medical apps during pregnancy in high-income countries, which may be an appropriate way of offering lifestyle intervention support to subfertile couples in the future. Lifestyle apps that aimed to improve health behavior, reduce gestational weight gain, and for smoking cessation were generally effective [40]. As such, internet-based technologies such as Smarter Pregnancy have the potential to answer some of these questions as well as raise awareness around the importance of pre-conception care when trying to conceive [21]. Moreover, cognitive behavior therapy or psychological support during fertility treatment is suggested to lead to significantly more viable pregnancies than routine care [41]. As such, pre-conception lifestyle interventions, including medical apps, have the potential to increase ongoing pregnancy rates and birth outcomes in subfertile couples [40].

We do not yet know if various lifestyle modifications such as smoking, alcohol, and nutrition have different effects that depend on the socioeconomic status, educational level, or social background of the couple trying to conceive. Recent analysis of Smarter Pregnancy suggests that the program has been more effective in women living in nondeprived neighborhoods, who were, however, less likely to complete the 24 weeks of coaching than women who lived in deprived neighborhoods [42]. Although subfertile couples seeking fertility treatment are often intrinsically motivated to make positive lifestyle changes to improve the likelihood of a successful pregnancy, the socioeconomic status of couples may impact lifestyle as well as their ability to change lifestyle habits.

### Conclusions

In summary, lifestyle is an important public health issue that has a significant and cumulative impact on fertility. Appropriate counseling could result in substantial reductions in the referrals for fertility investigations and treatments [27]. In order to maximize the pregnancy rates during fertility care, many subfertile couples could benefit from pre-conception lifestyle interventions delivered before fertility treatment. Although more research is needed regarding the use of internet- and app-based technologies for lifestyle and lifestyle interventions prior to fertility treatment, our results can be used to support subfertile couples, fertility care providers, and policy makers involved in public health to optimize clinical outcomes at affordable costs. Pre-conception lifestyle interventions are likely to be a cost-effective way of supporting subfertile couples trying to conceive. Further implementation of the app Smarter Pregnancy also designed for subfertile couples seeking fertility assistance is likely to be cost-effective and allow data on reproductive outcomes to be collected.

## Appendix

Multimedia Appendix 1 Table S1: Input parameters for specific lifestyle interventions per year.

Multimedia Appendix 2 Table S2: Input parameters – assisted reproductive technology costs, assumptions and fertility data from the Netherlands.

## Abbreviations

BMI: body mass index
IUI: intrauterine insemination
IVF: in vitro fertilization
ICSI: intracytoplasmic sperm injection
UPBEAT: UK Pregnancies Better Eating And Activity Trial
