# Supplementary material for: Pre-Conception Interventions for Subfertile Couples Undergoing Assisted Reproductive Technology Treatment: Modeling Analysis
Source: JMIR Mhealth Uhealth. 2020 Nov 23;8(11):e19570. doi: 10.2196/19570 (PMC7721553; doi:10.2196/19570)
Supplement: Multimedia Appendix 1 [file mhealth_v8i11e19570_app1.docx]

**Table S1. Input parameters for specific lifestyle interventions per year**

| **Variable (parameter)** | **Value** |
| --- | --- |
|  |  |
| Average total costs of fetal growth restriction [1] | €7,823 |
| Costs of *Smarter Pregnancy* for 2 years [1] | €119.80 |
| Standard risk of fetal growth restriction [2] | 8.6% |
| Chance of spontaneous pregnancy [3] | 32.5% |
| Relative increase in number of pregnancies as a result of *Smarter Pregnancy* *[4]* | 40% |
| Relative reduction in risk of fetal growth restriction as a result of *Smarter Pregnancy*: 30% [5, 6] | 30% |
| Delta costs fertility care (medication and direct medical costs) as a result of *LIFEstyle* [7]   - This is the cost difference between intervention and control group that did not receive lifestyle intervention | €940 |
| Average total costs in the case of gestational diabetes [8-11] | €6,626 |
| Average total costs in the case of hypertensive complications [10, 11] | €8,522 |
| Average total costs in the case of prematurity [4] | €8,563 |
| Costs of *LIFEstyle* for 2 years [7]   - Consultation costs: 30 minutes for a nurse were, €30.70, and for telephone consultation: €15.40 euro for 15 minutes. Over two years, participants could have 5 face to face consultations and 5 telephone consultations, so that is every 2.5 month a consultation | €246 |
| Risk of gestational diabetes after *LIFEstyle* [12] | 15% |
| Risk of gestational diabetes without *LIFEstyle* [12] | 19% |
| Standard risk of hypertensive complications [12] | 16.2% |
| Reduced risk of hypertensive complications as a result of *LIFEstyle* [12] | 3.76% |
| Standard risk of prematurity [12] | 14.40% |
| Reduced risk of prematurity as a result of *LIFEstyle* [12] | 2.95% |
| Effectiveness of LIFEstyle in reducing the risk of hypertensive complications and prematurity [12] | 38% |
| Relative increase of success after the 1st IVF attempt [13] | 21% |
| Relative increase of success after the 1st ICSI attempt [13] | 17% |
| Effectiveness of app [14] | 33% |
| Relative increase in clinical success rate IVF [15] | 19% |
| Number of started IVF cycles^a^ [16] | 6,509 |
| Number of started ICSI cycles^a^ [16] | 7,605 |
| Number of individuals per group counselling [15] | 15 |
| Percentage completers (counselling) [15] | 67.5% |

^a^ The number of women who started IVF or ICSI cycles in the Netherlands, the specific target group for our calculation for this intervention.

ART, assisted reproductive technology; IVF, *in vitro* fertilization; ICSI, intracytoplasmic sperm injection.

**References**

1. slimmerzwanger.nl. What does Smarter Pregnancy cost?. Available from <https://slimmerzwanger.hl/nl/wat-kost-het.php> Accessed August 17, 2018.

2. Foetale Groeibeperking Versie 2.1. Nederlandse Vereniging voor Obstetrie and gynaecologie. Available from: <http://www.med-info.nl/Richtlijnen/Gynecologie/Perinatologie/Foetale%20Groeibeperking.pdf> Accessed August 17, 2018.

3. NHG-Standaard Subfertiliteit. Dutch General Practitioners Association (Nederlands Huisartsen Genootschap). Available from <https://www.nhg.org/standaarden/volledig/nhg-standaard-subfertiliteit> Accessed August 18, 2018.

4. Vijgen SM, van der Ham DP, Bijlenga D, van Beek JJ, Bloemenkamp KW, Kwee A, et al. Economic analysis comparing induction of labor and expectant management in women with preterm prelabor rupture of membranes between 34 and 37 weeks (PPROMEXIL trial). Acta Obstet Gynecol Scand. 2014 Apr;93(4):374-81. PMID: 24392746. doi: 10.1111/aogs.12329.

5. Bouwland-Both MI, Steegers-Theunissen RP, Vujkovic M, Lesaffre EM, Mook-Kanamori DO, Hofman A, et al. A periconceptional energy-rich dietary pattern is associated with early fetal growth: the Generation R study. BJOG. 2013 Mar;120(4):435-45. PMID: 23194298. doi: 10.1111/1471-0528.12086.

6. Timmermans S, Steegers-Theunissen RP, Vujkovic M, den Breeijen H, Russcher H, Lindemans J, et al. The Mediterranean diet and fetal size parameters: the Generation R Study. Br J Nutr. 2012 Oct 28;108(8):1399-409. PMID: 22348517. doi: 10.1017/S000711451100691X.

7. van Oers AM, Mutsaerts MAQ, Burggraaff JM, Kuchenbecker WKH, Perquin DAM, Koks CAM, et al. Association between periconceptional weight loss and maternal and neonatal outcomes in obese infertile women. PLoS One. 2018;13(3):e0192670. PMID: 29590118. doi: 10.1371/journal.pone.0192670.

8. Koning SH, Hoogenberg K, Lutgers HL, van den Berg PP, Wolffenbuttel BH. Gestational Diabetes Mellitus:current knowledge and unmet needs. J Diabetes. 2016 Nov;8(6):770-81. PMID: 27121958. doi: 10.1111/1753-0407.12422.

9. Open data from the Dutch Health Authority. Nederlandse Zorgautoriteit. Available from <http://www.opendisdata.nl> Accessed August 13, 2018.

10. Vijgen SM, Koopmans CM, Opmeer BC, Groen H, Bijlenga D, Aarnoudse JG, et al. An economic analysis of induction of labour and expectant monitoring in women with gestational hypertension or pre-eclampsia at term (HYPITAT trial). BJOG. 2010 Dec;117(13):1577-85. PMID: 20840526. doi: 10.1111/j.1471-0528.2010.02710.x.

11. Lukassen HG, Schonbeck Y, Adang EM, Braat DD, Zielhuis GA, Kremer JA. Cost analysis of singleton versus twin pregnancies after in vitro fertilization. Fertil Steril. 2004 May;81(5):1240-6. PMID: 15136084. doi: 10.1016/j.fertnstert.2003.10.029.

12. Mutsaerts MA, van Oers AM, Groen H, Burggraaff JM, Kuchenbecker WK, Perquin DA, et al. Randomized Trial of a Lifestyle Program in Obese Infertile Women. N Engl J Med. 2016 May 19;374(20):1942-53. PMID: 27192672. doi: 10.1056/NEJMoa1505297.

13. Zitzmann M, Rolf C, Nordhoff V, Schrader G, Rickert-Fohring M, Gassner P, et al. Male smokers have a decreased success rate for in vitro fertilization and intracytoplasmic sperm injection. Fertil Steril. 2003 Jun;79 Suppl 3:1550-4. PMID: 12801558.

14. Gordon JS, Armin J, M DH, Giacobbi P, Jr., Cunningham JK, Johnson T, et al. Development and evaluation of the See Me Smoke-Free multi-behavioral mHealth app for women smokers. Transl Behav Med. 2017 Jun;7(2):172-84. PMID: 28155107. doi: 10.1007/s13142-017-0463-7.

15. Li J, Long L, Liu Y, He W, Li M. Effects of a mindfulness-based intervention on fertility quality of life and pregnancy rates among women subjected to first in vitro fertilization treatment. Behav Res Ther. 2016 Feb;77:96-104. PMID: 26742022. doi: 10.1016/j.brat.2015.12.010.

16. [www.novg.nl](file:///\\ferringworld.local\dfs\NL0\Cross-Org\VBHC\Fertiliteit\4%20-%20Patiëntuitkomsten\Publicatie%20business%20case%20leefstijl\www.novg.nl). IVF figures 2016: record number of children with small increase in treatment cycles and muliple births, Availbel from URL: <https://www.nvog.nl/actueel/ivf-cijfers-2016-recordaantal-kinderen-geringe-toename-behandelcycli-en-meerlingen/> Accessed September 11, 2018.
