# Supplementary material for: Pre-Conception Interventions for Subfertile Couples Undergoing Assisted Reproductive Technology Treatment: Modeling Analysis
Source: JMIR Mhealth Uhealth. 2020 Nov 23;8(11):e19570. doi: 10.2196/19570 (PMC7721553; doi:10.2196/19570)
Supplement: Multimedia Appendix 2 [file mhealth_v8i11e19570_app2.docx]

**Table S2. Input parameters – assisted reproductive technology costs, assumptions and fertility data from the Netherlands**

| **Author** | **Year** | **Country** | **Study population** | **Study design** | **Sample Size** | **Input parameter** |
| --- | --- | --- | --- | --- | --- | --- |
|  |  |  |  |  |  |  |
| Fiddelers, Dirksen [1] | 2009 | The Netherlands | Women seeking ART | Real-world cost analysis | Multiple sources (RCTs and non-RCTs, literature, and expert opinion) | Cost of IVF cycle: €4,184.22 |
| Fiddelers, Dirksen [1] | 2009 | The Netherlands | Women seeking ART | Real-world cost analysis | Multiple sources (RCTs and non-RCTs, literature, and expert opion) | Cost of 1 ICSI cycle: €4,752.02 |
| Dutch Health Authority [2] | 2018 | The Netherlands | NA | Real-world costs | Nation-wide | Cost of IUI: €745.00 |
| Lukassen, Schonbeck [3] | 2004 | The Netherlands | Women with singleton or twin pregnancies | Real-world costs | 279 women | Cost of standard pregnancy after IVF: €3,414 |
| NHG-Standaard Subferiliteit [4] | 2017 | The Netherlands | Real-world data | NA | NA | Prevalence of subfertile women 25–44 years: 2.2% |
| NHG-Standaard Subferiliteit [4] | 2017 | The Netherlands | Real-world data | NA | NA | Prevalence of subfertile men, 25–44 years: 0.7% |
| Dutch agency for Statistics [5] | 2017 | The Netherlands | Real-world data | NA | NA | Number of women, 25–44 years: 2,103,649 |
| Dutch agency for Statistics [5] | 2017 | The Netherlands | Real-world data | NA | NA | Number of men 25–44 years: 2,114,089 |
| Clinical data* | – | – | – | – | – | IVF: 24% |
| Clinical data* | – | – | – | – | – | IUI: 60% |
| Clinical data* | – | – | – | – | – | ICSI: 16% |
| Clinical data* | – | – | – | – | – | IVF: 1.5 |
| Clinical data* | – | – | – | – | – | IUI: 3.0 |
| Clinical data* | – | – | – | – | – | ICSI: 1.5 |

*Data based on clinical data from medical/fertility specialists from Erasmus MC University, who are members of the working group.

ART, assisted reproductive technology; IVF, *in vitro* fertilization; ICSI, intracytoplasmic sperm injection; IUI, intrauterine insemination; RCT, randomized controlled trial.

**References**

1. Fiddelers AA, Dirksen CD, Dumoulin JC, van Montfoort AP, Land JA, Janssen JM, et al. Cost-effectiveness of seven IVF strategies: results of a Markov decision-analytic model. Hum Reprod. 2009 Jul;24(7):1648-55. PMID: 19318703. doi: 10.1093/humrep/dep041.

2. Open data from the Dutch Health Authority. Nederlandse Zorgautoriteit. Available from <http://www.opendisdata.nl> Accessed August 13, 2018.

3. Lukassen HG, Schonbeck Y, Adang EM, Braat DD, Zielhuis GA, Kremer JA. Cost analysis of singleton versus twin pregnancies after in vitro fertilization. Fertil Steril. 2004 May;81(5):1240-6. PMID: 15136084. doi: 10.1016/j.fertnstert.2003.10.029.

4. NHG-Standaard Subfertiliteit. Dutch General Practitioners Association (Nederlands Huisartsen Genootschap). Available from <https://www.nhg.org/standaarden/volledig/nhg-standaard-subfertiliteit> Accessed August 18, 2018.

5. Statistics Netherlands (CBS). Dutch Agency for Statistics. Available from URL: <https://opendata.cbs.nl/> Accessed November 9, 2018.
